# Evolution: like any other science it is predictable

**DOI:** 10.1098/rstb.2009.0154

**Published:** 2010-01-12

**Authors:** Simon Conway Morris

**Affiliations:** Department of Earth Sciences, University of Cambridge, Downing Street, Cambridge CB2 3EQ, UK

**Keywords:** evolution, convergence, frogs, theropods, *E. coli*, viruses

## Abstract

Evolutionary biology rejoices in the diversity of life, but this comes at a cost: other than working in the common framework of neo-Darwinian evolution, specialists in, for example, diatoms and mammals have little to say to each other. Accordingly, their research tends to track the particularities and peculiarities of a given group and seldom enquires whether there are any wider or deeper sets of explanations. Here, I present evidence in support of the heterodox idea that evolution might look to a general theory that does more than serve as a tautology (‘evolution explains evolution’). Specifically, I argue that far from its myriad of products being fortuitous and accidental, evolution is remarkably predictable. Thus, I urge a move away from the continuing obsession with Darwinian mechanisms, which are entirely uncontroversial. Rather, I emphasize why we should seek explanations for ubiquitous evolutionary convergence, as well as the emergence of complex integrated systems. At present, evolutionary theory seems to be akin to nineteenth-century physics, blissfully unaware of the imminent arrival of quantum mechanics and general relativity. Physics had its Newton, biology its Darwin: evolutionary biology now awaits its Einstein.

## 1. Introduction

Oxymoronically, the sheer obviousness of organic evolution can only explain the many quotations that declare the self-evident. Consider, for example, Thomas Henry Huxley's post-1859 exclamation of ‘How extremely stupid not to have thought of that!’ (Huxley 1900, vol. 1, p. 170), or more recently perhaps Richard Dawkins' somewhat hypothetical question posed by visiting extraterrestrials as to whether we had yet stumbled on the Darwinian formula (Dawkins 1989, p. 1). Both, of course, miss the point. Whatever Huxley's talents, he had not the remotest chance of formulating a workable hypothesis for organic evolution. Indeed, even after the publication of Darwin's *Origin*, he kept on missing the point (Desmond 1998). So too I suspect any extraterrestrial tourists are more likely to enquire whether we have yet dropped the habit of killing millions of innocents on the basis of truly daft ideologies, as likely as not based on some mutant French *philosophie*. And should the conversation stray to science, they might politely enquire as to the current progress in our understanding, say, of quantum entanglement.

Yet, if evolution is glaringly obvious, why is it not only greeted with growing hostility, but the siren-call of anti-evolutionary dogma, notably ‘intelligent design’, remains a rallying point to individuals that in any other respect fail to manifest any obvious sign of mental instability? The reasons, of course, are complex and so far as the explication (and defence) of the science of evolution is concerned, it can hardly be assisted by those who ironically treat it as a religion (Midgley 1985). I wonder if paradoxically the difficulty stems from Darwin himself. Curiously, it is seldom appreciated that whatever else his masterpiece (Darwin 1860) set out to achieve it was at heart an exorcism of William Paley (Conway Morris 2009). With consummate skill, and in striking contrast to the belligerent and graceless rhetoric of some of his intellectual descendants, he systematically dismantled Paley's creationism. But 150 years on the message has evidently failed to sink in.

While the aim of this paper is, I hope, straightforward, it is also designed to point to some unfinished business. Of course, and as already indicated, the reality of evolution is not in dispute. Nor is the Darwinian formulation, for which the evidence is overwhelming. The question quite simply is whether the theory is complete. At heart are the questions as to what life is itself and the nature of the organizational principles that might underpin it. The first topic was most famously articulated by Schrödinger (1948), and continues to receive attention, albeit at times in a somewhat desultory (if not unfocused) fashion. But the question is of central importance and a recent and highly germane articulation is given by Macklem (2008). As he points out, life seems to occupy a very precise zone (indeed, I suspect a gossamer-like tight-rope) perched between systems that are either very highly ordered (crystalline) or largely chaotic and subject to recurrent instabilities. As he concludes, ‘understanding life requires knowledge of how the design of living creatures and emergent phenomena, appearing spontaneously in self-ordered, reproducing, interacting, energy-consuming, nonlinear, dynamic ensembles makes us what we are. I believe this will be the next biological revolution’ (Macklem 2008, p. 1846). This writer also emphasizes the central role of emergence in biological systems. Together with the related topic of self-organization, these concepts can be melded with the currency of evolution in the form of developmental constraints (the role of which may be exaggerated) and epigenetics to suggest that indeed something is missing in the Darwinian synthesis.

Here, I will suggest that one central tenet of the current neo-Darwinian synthesis, that evolution is for all intents and purposes open-ended and indeterminate in terms of predictable outcomes, is now open to question. Thus, not only is life suspended between permanently uninhabitable regions that are either locked into crystalline immobility or in continuous and chaotic flux, but that the lines of evolutionary vitality thread through a landscape that leaves evolution with surprisingly few choices. The basis of this view relies on the phenomenon of evolutionary convergence. This concept is, of course, not only entirely familiar to evolutionary biologists, but also provides some of the strongest arguments in favour of adaptational explanations. However, much less appreciated is the ubiquity of this convergence, with examples spanning the entire biological hierarchy from molecules to social systems and cognitive processes. In support of this thesis, which I explore at far greater length elsewhere, I briefly touch on (i) what, if any, key steps in the evolution of life are entirely fortuitous and (ii) what, if any, biological innovations are unique?

## 2. Are the key steps in evolution fortuitous?

If there is a fatal flaw in the argument for evolutionary inevitabilities, if not a determinism, then it is in the widely accepted proposition that certain key transitions in the history of life are the result of effectively fortuitous sets of evolutionary events that in combination are so improbable as to render the process fundamentally unpredictable. That evolution is not utterly random is evident from the ubiquity of homoplasy, at least within clades that encompass lower parts of the taxonomic hierarchy. The question, however, is does this principle extend to the major divisions of life? No definitive answer can yet be given, not least because the origins of the great majority of major groups are shrouded in obscurity, although jointly molecular data and the fossil record continue to make major assaults on this citadel of ignorance. One can, moreover, point to the particular examples that, I suggest, point to a more general principle.

### (a) Frogs and theropods

Consider, for example, the seemingly arcane area of frog ecomorphs. As befits an evolutionary laboratory, the frogs of Madagascar show a series of adaptive radiations, with the occupation of habitats as diverse as burrowing, as well as dwelling in trees, rocks and torrential streams. These ecomorphs find a series of striking convergences (Bossuyt & Milinkovich 2000) with the frogs of Asia (principally India), and so too in this latter region there are further episodes of parallel evolution (e.g. independent development of fangs). The comparisons between Madagascan and Asian frogs are all the more striking because they extend to the larval forms, but there is one striking omission. Thus, in Asia there is no counterpart to the iconic poisonous mantellids. So, the principle of the repeatability of evolution fails at the first hurdle? Not quite, because the mantellids display a series of striking convergences with the neotropical dendrobatids (poison-arrow frogs; e.g. Clark *et al*. 2005).

The question, therefore, is how far does this principle extend? To be sure, the evidence is that both frogs and amphibians (and indeed vertebrates and animals) are monophyletic, yet in each and every case the assemblage of the body plans (at whatever taxonomic level; Conway Morris 2002) reveals a complex (and often controversial) story of stem groups typified by striking parallels in evolutionary directionality. In addition, although widely remarked upon the general observation that early in the evolution of a group there is very often a mélange of ‘unexpected’ features, leading to repeated remarks of ‘bizarre’ morphologies and ‘problematic relationships’, is actually one of profound evolutionary importance. A recent example involves a new reptile close to the origin of the birds (Zhang *et al*. 2008), which in turn underlines some important generalizations. Thus, when we consider the origin of the birds, the story of *Archaeopteryx* and its theropod connection, not to mention the spectacular evidence of subsequent bird evolution in the Cretaceous (e.g. Ji & Ji 2007; Zhou *et al*. 2008), needs no emphasis. Perhaps, less well appreciated is that within this group the Late Cretaceous genus *Rahonavis ostromi*, initially interpreted as a primitive bird (Foster *et al*. 1998*a*,*b*; see also Zhou 2004), and indeed coexisting in Madagascar with genuine birds (Foster *et al*. 1996), is now placed in the dromaeosaurids and is close to such genera as *Buitreraptor* and *Unenlagia* (e.g. Makovicky *et al*. 2005; Senter 2007). So too independently in this group we see the evolution of the extraordinary four-winged *Microraptor gui* (Xu *et al*. 2003; see also Zhou 2004), although in this case the capacity for aerial excursions more probably involved an undulatory gliding rather than powered flight (Chatterjee & Templin 2007; but see Xu *et al*. 2005).

**Figure 1.**
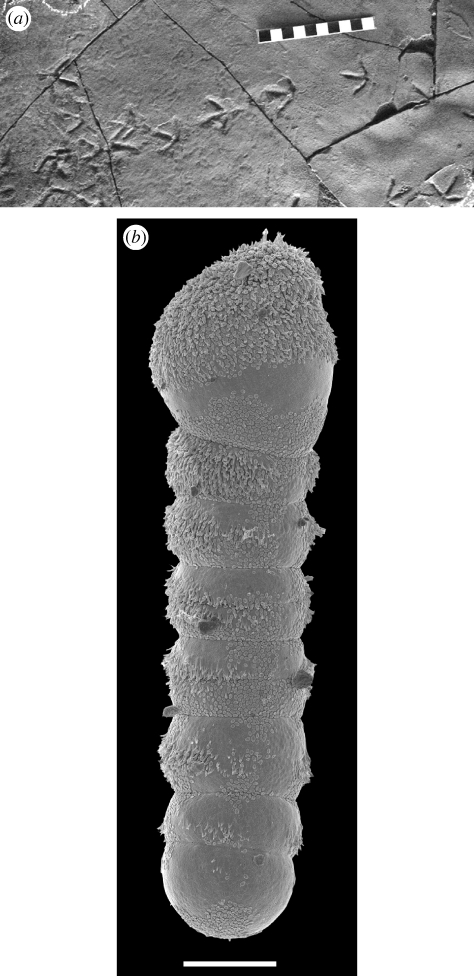
Two ‘unexpected’ examples of convergent evolution. (*a*) Trackways from the Santo Domingo Formation (Late Triassic–Early Jurassic) of Argentina that are interpreted as representing the activities of some sort of theropodian avialian (‘bird’), and possibly convergent to other flying theropods. Centimetric scale bar. Picture courtesy of Ricardo Melchor (Universidad Nacional de la Pampea, Argentina). (*b*) A protistan convergence. The dinoflagellate *Haplozoon praxillellae*, an intestinal parasite of polychaete worms that has converged on a cestode-like bodyform, including attachment structures, strobilation and a hairy covering. Scale bar, 10 mm. Picture courtesy of Brian Leander (University of British Columbia).

These observations need some qualifications. Thus, the identification of feathers on the legs of *Archaeopteryx* (Christiansen & Bonde 2004; Longrich 2006), as well as more derived birds (Zhang & Zhou 2004), and even maniraptorian dinosaurs (Xu & Zhang 2005; see also Zhang *et al*. 2008), may have a bearing (in the last case) on their pre-adaptation for aerial activity and also the aerodynamic origins and subsequent performance once aloft. However, not only are a variety of integumentary structures known (e.g. Zhang *et al*. 2006), but in the case of some examples, such as *Velociraptor*, the forearms evidently carried feathers but the animal was far too large to fly (Turner *et al*. 2007). It is also evident that although the small size necessary for an aerial mode of life was ancestral in this group, such miniaturization was achieved *before* the capacity for flight and there is also a series of striking parallels towards gigantism (Turner *et al*. 2007). Finally, we need to remind ourselves that evidence from trackways suggests that the diversity of this group is not completely understood (Li *et al*. 2008), while intriguingly bird-like foot-prints (figure 1*a*) from the Late Triassic (or Early Jurassic) hint at a much earlier group of avian-like theropods (Melchor *et al*. 2002; De Valais & Melchor 2008). Indeed, so striking are these imprints that point not only to the various styles of walking, but also evidently probing and alighting (as seen in living shore-birds), that the proposed age needs assessment (Genise *et al*. 2009). It is, however, based on apparently reliable radiometric and palaeobotanical evidence (De Valais & Melchor 2008).

In the theropods, therefore, flight evolved independently at least two times, and quite conceivably four times. Nor would I be surprised if this total was to grow. One of the many disadvantages of the cladistic methodology, apart from its profound lack of interest in functional integration (and too often ontogenetic trajectories) and a preference for a bizarre atomism, is that it is largely resistant to the concept of evolution being a highly dynamic process where we see not only mosaic evolution (e.g. Ji & Ji 2007), but also the repeated acquisition and loss of features. This point is well made by Kurochkin (2006). Thus, one can hardly be surprised that something very like a bird evolved multiple times among the theropods: both bipedality and integumentary modifications are vital pre-adaptations. But do we not face an indefinite regress of evolutionary cause and effect? All ‘birds’ arise from within the theropods, but how probable is a theropod-like construction *per se*? An important clue comes from the archosaurians, a more primitive group of reptiles that flourished in the Triassic. Here, in the archosaur *Effigia*, we see a series of striking convergences with not only the theropods, but even the coelurosaurians and in turn the ornithomimosaurians (Nesbitt & Norell 2006; Nesbitt 2007). *Effigia* is most closely related to *Shuvosaurus*, and this latter genus had previously been interpreted as a dinosaur. The dangers of convergence are spelt out by Parker *et al*. (2005) in their description of another archosaur *Revueltosaurus*. This is simply because with the recovery of more complete skeletons it has become evident that isolated teeth (the common currency of much of vertebrate palaeontology), which had routinely been assigned to Late Triassic dinosaurs, are just as likely to derive from convergent archosaurian taxa.

In fact, it now transpires that there are a series of striking convergences between archosaur and dinosaur morphs that go well beyond the theropod-like forms mentioned above. Thus, among the archosaurs, we see both carnosaur-like and ankylosaur-like forms, which in a number of cases were previously thought to be dinosaurs. Molnar (2008, p. 586) extended this list and made the important point that ‘far from being a mere curiosity, the Triassic instances (of convergence) affect our whole understanding of the evolutionary origin of important components of the Mesozoic tetrapod faunae, and provoke questions of why animals seemingly effectively occupying certain ecological niches were somehow replaced by phyletically distinct forms sufficiently similar to be mistaken for one another’. So, it seems that something very like a dinosaur is very much on the evolutionary cards. From this perspective, the important insights on the question of competitive superiority of the dinosaurs as against the archosaurs (Brusatte *et al*. 2008) still need to be seen in the wider context of the likelihood of a dinosauromorph (or indeed a reptiliomorph, a tetrapodiomorph, and so on).

### (b) Carboxysomes and viruses

**Figure 2.**
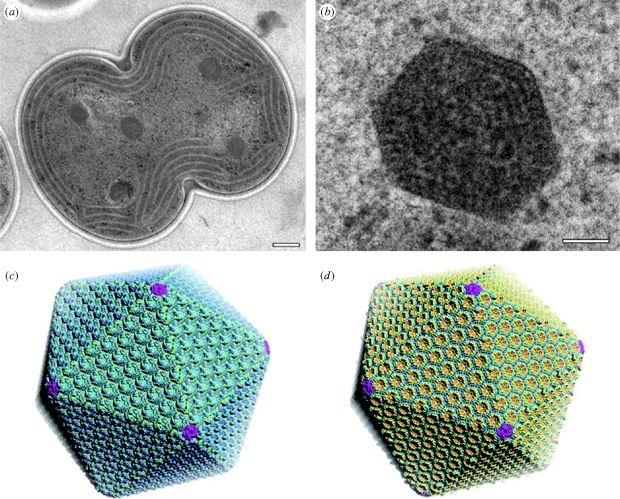
Another ‘unexpected’ example of convergent evolution. Carboxysomes of bacteria that are strikingly similar to the protein coats of viruses, but are independently evolved. Upper, transmission electron micrographs of carboxysomes in a cyanobacterium (*Synechocystis*). (*a*) Entire cell in the process of dividing, five polyhedral carboxysomes are visible. Scale bar, 200 nm; (*b*) individual carboxysome. Scale bar, 50 nm. Reproduced from fig. 1*a*,*b* in Kerfeld, C.A., Sawaya, M.R., Tanaka, S., Nguyen, C.V., Phillips, M., Beeby, M. & Yeates, T.O. 2005 Protein structures forming the shell of primitive bacterial organelles. *Science* 309, 936–938; with the permission of AAAS and the authors. (*c*) and (*d*) show the alternative models for the carboxysome shell. Each is based on a shell constructed of 740 hexameral units and 12 pentamers, and the two models (*c*,*d*) differ in terms of the orientation of the hexamers. Reproduced from fig. 3*d* in Tanaka, S., Kerfeld, C.A., Sawaya, M.R., Cai, F., Heinhorst, S., Cannon, G.C. & Yeates, T.O. 2008 Atomic-level models of the bacterial carboxysome shell. *Science* 319, 1083–1086; with the permission of AAAS and the authors.

Birds, theropods, dinosaurs and archosaurs may be, respectively, instructive in terms of convergences, and indeed I believe they point to a general, but neglected, principle. The details remain to be tested, but here I offer an outline as to a series of convergences associated with some of the major transitions in evolution that indicate that in each case the step was very probable, if not inevitable. Consider first the viruses. While the notion that they may be the most primitive forms of life has been largely abandoned, they do provide a useful proxy for the minimum desiderata for an organism. One of the defining characteristics of a virus is, of course, the highly organized protein coat. When we turn to the micro-compartments of a number of eubacteria, and especially the carboxysomes (figure 2), they too build a polyhedral protein coat in a strikingly similar fashion (Cannon *et al*. 2001; Kerfeld *et al*. 2005; Bobik 2006). To be sure the carboxysomes are not icosahedral and the coat itself is thinner (perhaps because of its organelle-like status; see Tsai *et al*. 2007), but the tightly packed hexameral arrangement evidently forms by self-assembly (Yeates *et al*. 2007), and the striking similarity between carboxysomes and viral coats has been repeatedly stressed. And with respect to viruses themselves, we see striking examples of convergence (e.g. Bull *et al*. 1997; Cuevas *et al*. 2002), no small matter given their role in disease (e.g. De Lamballerie *et al*. 2008; Kryazhimskiy *et al*. 2008). However, in terms of viral convergence arguably the most fascinating examples involve the giant DNA viruses, best known in the form of the mimivirus (e.g. Suhre 2005). These viruses are effectively re-inventing themselves as true organisms, with genomes substantially larger than some bacteria, and driven by both gene duplication and lateral transfer from their hosts. Significantly, however, the two principal groups (T4 and NCLDVs) are strikingly convergent in not only the methods of genome increase, but also the locations of the laterally transferred material in the viral genome (Fileé & Chandler 2008).

### (c) Bacteria: re-running the tape

As already indicated, it is not suggested that bacteria are derived from viruses (or viral coats from carboxysomes), but these examples are indicative that the evolution of viruses may be more constrained than might be imagined. So too among the prokaryotes, we find many striking convergences not only within the archaea and eubacteria, but also more significantly between these two groups. One of the most interesting, and especially important because of its misappropriation by the proponents of the scientific fiction referred to as ‘intelligent design’, is the independent evolution of the flagellar motor in either bacterial group (e.g. Thomas *et al*. 2001; Trachtenberg *et al*. 2005). Among the other convergences that occur between the archaea and the ubacteria, particularly striking examples can be found among the extremophiles, notably the halophiles (e.g. Mongodin *et al*. 2005; Paul *et al*. 2008) and thermophiles (e.g. Lin 2008; Puigbò *et al*. 2008). Given such extremophiles are a major focus of attention for what may be typical extraterrestrial environments (e.g. Tosca *et al*. 2008), and recalling that thermophiles can flourish at 122°C (Takai *et al*. 2008) and in supersaturated brines (Mongodin *et al*. 2005), then these convergences may confer an unexpected predictability in terms of remote microbial biospheres.

The very rapid rates of reproduction and the ease of maintaining a series of isolated clones, not to mention the capacity to create a complete ‘fossil record’ by the expedient of freezing the sample of a given population, is our best approximation of re-running the tape of life, at least in a microbial context. This has been achieved with spectacular success by Rich Lenski and colleagues and in *Escherichia coli* has revealed a number of striking instances of convergent evolution (e.g. Travisano *et al*. 1995; Cooper *et al*. 2003). By no means are all trends convergent, and more importantly there are striking instances where historical contingency evidently underlies the emergence of a key innovation. One such example is the capacity of *E. coli* to use a citrate substrate (Blout *et al*. 2008). By way of background, it should be explained that the experiment depends on 12 independent lineages of *E. coli*, initially identical, which over tens of thousands of generations have evolved and so explored a large mutation space. At first sight the unexpected emergence of a citrate capacity is exactly the sort of test case that would appear to torpedo the entire thesis of this paper. A closer examination actually reveals the exact reverse. Thus, while the mutations that enabled this experimental population of *E. coli* to use a citrate substrate after more than 30 000 generations do appear to be genuinely fortuitous, we need first to recall that *E. coli* is very unusual in being unable under anaerobic conditions to employ citrate (and as such is a convenient way to identify this bacterium in a medical context). The vast majority of other micro-organisms can employ citrate (e.g. Bott 1997; Polen *et al*. 2007), and more significantly not only can *E. coli* do the same in the natural environment but it also does this by virtue of plasmids (e.g. Ishiguro *et al*. 1988). The origin of these plasmids appears to be obscure, but the actual citrate carrier (CitT) belongs to a novel eubacterial transporter family (Pos *et al*. 1998). Not only have a wide range of these citrate carrier plasmids been identified (Sasatsu *et al*. 1985), but looking across a wider range of bacteria these two types of plasmids fall into at least two distinct classes in terms of sequence (Shinagawa *et al*. 1982).

Interesting as the results of Blout *et al*. (2008, p. 7905) most certainly are, when the declared title is ‘Historical contingency and the evolution of a key innovation’ and their paper concludes by proclaiming that ‘our study shows that historical contingency can have a profound and lasting impact under the simplest, and thus most stringent, conditions in which initially identical populations evolve in identical environments. Even from so simple a beginning, small happenstances of history may lead populations along different evolutionary paths. A potentiated cell (for citrate utilization) took the one less travelled by, and that has made all the difference’ then we need to register a mild protest. To be sure this stirring rhetorical mélange of Charles Darwin and Scott Peck (if not Robert Frost) certainly applies to this experimental population of *E. coli* but let us take the wider view. Thus, the ubiquity of citrate utilization and the independent employment of plasmids with citrate carriers suggest that the contingency identified by Blout and co-workers is genuine, but of only parochial relevance.

## 3. Key steps

These few examples of evolutionary convergence are only indicative of what I suspect will be a ubiquitous phenomenon, but of course the origins of the archaea and eubacteria are still shrouded in mystery. So too is the nature of the first eukaryote, and while there is now general agreement that it is effectively a chimaera of the two prokaryotic lineages (e.g. Yutin *et al*. 2008), it is certainly possible to argue that the eukaryotic condition is also more or less fortuitous. Nevertheless, there are some pointers in the opposite direction. For example, it is now clear that bacteria possess a cytoskeleton, including tubulin, actin and intermediate filaments (e.g. Graumann 2007; Pogliano 2008). Although showing no sequence similarity to the eukaryotic equivalents, at the moment the consensus is that these proteins are homologous. Nevertheless, their diverse functionality in the bacteria reflects the evolutionary versatility of these cytoskeletal elements. And again, we find the echoes of eukaryotic potentiality in the bacteria. Thus, in the famous magnetotactic bacteria, whose employment of magnetic minerals is convergent (DeLong *et al*. 1993), the enclosing magnetosomes employ actin and this membranous organization recalls, of course, a type of organellar construction. Another key aspect of eukaryotic construction is the complex internal membranes, exemplified by the Golgi apparatus and endoplasmic reticulum. But again in bacteria we find intriguing parallels that involve both the attachment of ribosomes (Herskovits *et al*. 2002) and what appears to be the budding of intracellular vesicles (Rachel *et al*. 2002). From these perspectives, the emergence of a eukaryotic form does not seem so improbable. Nor does the independent evolution of multicellularity in the prokaryotes, of which the *Eudorina*-like arrangement in some magnetotactic bacteria is arguably the most striking (Keim *et al*. 2004).

### (a) Endosymbiosis and endocytosis

If there is one aspect of the origin of eukaryotes that is not controversial, then it is the endosymbiotic derivation of the chloroplasts and mitochondria. In the latter case, current evidence suggests that this was a unique event, the ur-mitochondrion deriving from an *α*-proteobacteria similar to the extant rickettsialids (Fitzpatrick *et al*. 2006). However, as these authors point out, the genomic diversity of this group is still poorly known. Given the drastic erosion of the genome in both the mitochondria and independently in the pathogenic rickettsialids, and in the former case the intense selective pressure to export as many genes as possible in the face of the production of oxidative free radicals (e.g. Allen 2003), then it would be quite possible that the convergence of function has obliterated mitochondrial polyphyly, even among the *α*-proteobacteria.

And the same may apply to the chloroplasts, where some evidence for a polyphyletic origin exists (Stiller 2003; Stiller *et al*. 2003; but see also Palmer 2003). So, while the evidence strongly points to the chloroplasts being derived from the cyanobacteria, and most probably those with heterocysts (Deusch *et al*. 2008), various workers have stressed that the sampling of extant cyanobacterial genomes is by no means complete and that extrapolation to ancestral forms living perhaps 2 billion years ago is not straightforward (Larkum *et al*. 2007). Combine this with the ever present difficulties of phylogenetic reconstruction, not least long-branch attraction, then the monophyly of chloroplasts (and mitochondria) may well be only a default position. In fact, this is only one example of a general problem in evolution, which in a nutshell has to decide if the obvious differences are the result of (unremarkable) divergence as against an over-looked polyphyly. As Howe *et al*. (2008) stressed, the wielding of Ockham's razor effectively pre-disposes the conclusion to monophyly.

In the case of the chloroplasts, a further reason for caution are the various instances of algal endosymbiosis, of which one of the most striking is the incorporation of a cyanobacterium (quite separate from the chloroplasts) as a quasi-plastid in the amoeba *Paulinella* (Marin *et al*. 2005; Archibald 2006; Rodriguez-Ezpeleta & Philippe 2006; and for a comparable case in a diatom, see Prechtl *et al*. 2004). In conclusion, the multiple evolution of at least the chloroplasts appears conceivable. It is also worth remembering that despite their respective roles in oxidative metabolism and photosynthesis, mitochondria and chloroplasts have striking similarities in the adaptations of their electron transport systems to endosymbiosis (Berry 2003), and as with a number of other molecular pathways one can enquire just how many alternatives are viable.

### (b) Protistans

Within the eukaryotes, we also encounter convergences that at first sight are decidedly puzzling because they clearly involve the re-invention of a structure that has already evolved in the common ancestor and so in principle is already available. One of the most striking examples comes from the ciliates where there is compelling evidence for the independent evolution of an important part of cell machinery involved with transport in the form of secretory vesicles and known as dense core granules (Elde *et al*. 2007). The evidence (Elde *et al*. 2005) is based on the independent evolution of the protein machinery, notably GTPases known as dynamins, and Nels Elde and colleagues raise the tantalizing possibility that ‘certain cellular pathways might be more prone to convergent evolution than others’ (Elde *et al*. 2007, p. 162) and they continue this important speculation to the effect that possibly ‘the capacity to make granules was inherent in the basic organization of the Golgi complex and TGN [*trans*-Golgi network]’. To speak of inherency in evolutionary circles is certainly heterodox, not least because it might point to the Darwinian process being far more predictable than customarily perceived, but as I have argued elsewhere (Conway Morris 1998, 2003), inherency is widely overlooked in an evolutionary context.

Another valuable service Elde *et al*. (2007) perform is to warn us against the uncritical reliance of assuming that similar structures are necessarily so because of common descent. This may be the appropriate default position, but one major contribution of the molecular revolution is to show repeatedly that similarity does not automatically equate with homology. Very many examples could be given (Conway Morris 2003), but among the most startling concerns the recognition that in the insects the olfactory transduction proteins (e.g. Benton 2006) follow the classic seven-helices transmembrane arrangement exemplified by the opsins, but the former clearly have a completely separate origin (Sato *et al*. 2008; Wicher *et al*. 2008). Given these proteins are the cheek-by-jowl (or the nearest arthropodan equivalent) to the visual opsins, and given also that in other animals all the transduction proteins (including opsins) appear to belong to a single large family (G-protein coupled receptors), it is curious that in the case of the insects they have re-invented the molecular wheel (and, of course, the overall configuration of the olfactory process is itself also strikingly convergent with that found in the vertebrates; e.g. Eisthen 2002; Ache & Young 2005; Kay & Stopfer 2006). But far more significant is that at least on this planet, and at least among the animals, the transduction of *any* sensory data will look to the same molecular solution.

Space does not allow a similar set of discourses on the likelihood or otherwise of key steps within the eukaryotes also being effectively inevitable, although there is certainly a striking evidence for convergence among the protistans (e.g. Leander 2008), not only in terms of recurrent architectures but also in terms of more specific instances. Particularly intriguing examples are found among the dinoflagellates. Thus, some show intriguing convergences with the ciliates (Yukubi & Leander 2008), but even more notable are the warnowiids with a camera-eye (e.g. Couillard 1984), which like the related polykrikids (Westfall *et al*. 1983) have also evolved cnidarian-like nematocysts (Greuet 1971) that are used to capture prey (Matsuoka *et al*. 2000). Other dinoflagellates are parasitic, and interesting as the convergences are among some fish ectoparasites (Levy *et al*. 2007), even more remarkable is the intestinal parasite (of a polychaete worm), known as *Haploxoon praxillellae* (figure 1*b*). This dinoflagellate has re-invented itself as a tapeworm, complete with attachment structures, segmental strobila terminating in sporocytes that break away and even strikingly similar surface ornamentation of microtrich-like structures (Leander 2008; Rueckert & Leander 2008).

### (c) The roads to multicellularity

What of more complex eukaryotes, especially those that are multicellular? Here too some more general principles may be instructive. Thus, it is important to emphasize that even if the basic molecular machinery has evolved at an early stage, the ways in which it is deployed in different groups may vary but, nevertheless, the independent duplications as paralagous genes may well lead to convergences, as, for example, in the vital process of membrane trafficking (Dacks *et al*. 2008). Of particular interest in this context are the SNAREs (*N*-ethylmalemide-sensitive factors attachment protein receptors). These play a central role in vesicle transport and membrane interactions. Not surprisingly, they are ubiquitous among eukaryotes, and even in the ancestral form are inferred to have shown considerable elaboration (Kloepper *et al*. 2007). Independently, in various eukaryotic lineages, the SNAREs have both diversified and undergone duplications, but there is a particularly intriguing correlation between the expansion of this gene family and the appearance of multicellularity (or other increases in organismal complexity; Dacks & Field 2007; Sanderfoot 2007; Kloepper *et al*. 2008).

To be sure the correlation is not absolute, in as much as single-celled protistans can have a very large number of SNAREs, while the fungi have a relatively limited number (Kloepper *et al*. 2007). However, as these authors point out, the large number of SNAREs in *Paramecium* is less surprising given ‘its stunningly complex subcellular organization’ (p. 3467), while the fungal data draw on two ascomycetes (yeast and *Neurospora*). In addition, Kloepper *et al*. (2007) included the oomycete *Phytophthora*, but this is now well known to be convergent with the fungi (Money *et al*. 2004), although in passing we should note that there is evidence that this convergence is unusual because in part it arises from lateral gene transfer (LGT) from ascomycete fungi to oomycetes. The identified genes, however, are involved principally with osmotrophy (Richards *et al*. 2006) and to date there is no evidence for LGT of the SNARE genes. In the case of those fungi which are histologically complex, such as *Sphaerobolus* (Webster & Weber 2007), it would be interesting to know if the diversity of SNAREs has increased. If so, this would support Sanderfoot's (2007) suggestion of a correlation between gene duplications of SNARE genes and the emergence of multicellularity, at least in the green plants.

The case of the animals, in which we may take at least a parochial interest, is particularly problematic at present. Thus, so far as the fossil record is concerned, the Ediacaran assemblages appear to offer some tantalizing insights into the diversity of early metazoans (and quite possibly other distantly related macroscopic groups), but to date they give no clues as to the transition from more primitive forms. Molecular phylogenies are scarcely more helpful because, while there is strong evidence for the protistan choanoflagellates being the sister group of animals (e.g. Carr *et al*. 2008), neither they nor related groups that include corallochyteans, ichthyosporeans and ministeriids (e.g. Steenkamp *et al*. 2006) give many clues as to how the transformation to animals might have been achieved. Certainly, the prior existence of genes linked to cell adhesion and signalling in choanoflagellates (King *et al*. 2008; Ruiz-Trillo *et al*. 2008) points to the pre-adaptations for multicellularity. Note, however, that in at least one choanoflagellate, the unicellular(!) *Monosiga*, the tyrosine kinase signalling apparatus is not only far more diverse than any metazoan (Manning *et al*. 2008, p. 9678), but as the investigators note this network reveals ‘several common themes that suggest convergent evolution and a limited set of recurring molecular themes favoured by signalling pathways’.

### (d) Homeotic convergences

Perhaps, one day, the entire molecular and morphological transition from protist to animal will be available, but if we enquire what fundamentally is required to make an animal among the most important presumably are: homeotic genes, structural molecules such as collagen, muscles for movement and nerves for the rapid propagation of information. Once again, it is difficult to see what might have prevented them from evolving. Thus, the homeodomain (HD) proteins go deep into eukaryotic history, and their presence in metazoans, fungi, *Dictyostelium* and plants points to a role in the evolution of multi-cellularity. Despite this striking association, Derelle *et al*. (2007) also argued that the last common ancestor of all eukaryotes possessed at least two types (TALE, non-TALE) of HD protein and, echoing the story of the SNARE genes, suggest that the rounds of duplication occurred independently. This leads them to ‘suggest that the eukaryotes as a whole are pre-adapted for multicellularity’ (Derelle *et al*. 2007, p. 217). In the context of evolutionary likelihoods, if not inevitabilities, it is also important to note that striking structural analogues to the HD proteins occur in the prokaryotes (Treisman *et al*. 1992; Kant *et al*. 2002). The independent emergence of more complex homeotic systems does not seem that improbable.

Moreover, the functional convergence of systems that serve to control cell proliferation and differentiation in plants and animals (respectively, GEM and geminin), with important implications for the evolution of complex multicellularity (Cara & Gutierrez 2007), again points to likelihoods rather than accidents. Unfortunately, to date, the origin of homeobox systems in the animals, such as the canonical Hox clusters, is obscure, not least because there is no evidence for either these or the other genes central to metazoan body plans occurring in even their sister group, the choanoflagellates (King *et al*. 2008, p. 787). Indeed, as these authors remark, both the invention of such genes and their integration to an already existing and complex network of signals and regulatory pathways ‘remain mysterious’. In at least one respect, however, the choanoflagellates are helpful because there is genomic evidence for the protein collagen (King *et al*. 2008), typically with repeated amino acid (aa) triplets (glycine–proline–another aa) and in all metazoans a key structural component. Moreover, given the collagen's essential reliance on the amino acids proline and hydroxyproline, disposed in a triple helix, its evolution does not seem to entail intractable steps. Indeed, a prokaryotic collagen-like protein (with multiple XXG repeats, typically proline–threonine–glycine) occurs in the outer wall of the anthrax spore (Daubenspeck *et al*. 2004).

So far as other diagnostic components of the animals are concerned, the origin of muscles seems more than probable given the ancient origins of the myosin molecular motors upon which they are based (e.g. Richards & Cavalier-Smith 2005; Foth *et al*. 2006). As with the SNAREs and HD proteins, we see a fascinating series of divergences for an extraordinary variety of functions that require molecular motors in the context of cellular transport and transduction (e.g. Thompson & Langford 2002). Also, parallel to the SNAREs and HD proteins, the molecular evidence concerning myosin evolution supports massive diversification (and convergences) but a single origin. Nevertheless, any familiarity with the recurrent molecular solutions to particular functional challenges (as might be the case in vesicle transport and membrane activity, DNA binding and actin-based motors) means that if any of these systems actually transpired to be convergent I will not be unduly surprised (but others will be).

### (e) An inevitable nervous system

Just as the present evidence indicates a monophyletic origin for animal musculature, so too the nervous system is restricted to the eumetazoans. Once again, however, we can see significant precursors that point to a deeper inevitability. Thus, it is not particularly surprising to find among the sponges, which evidently lack any nervous tissue, a series of proteins that (with some notable absences) are otherwise central to the post-synaptic configuration in higher animals (Sakarya *et al*. 2007). These proteins have been identified in a distinctive group of flask-shaped cells and the fact that they display molecular mechanisms that are also the hallmark of neurogenesis (Richards *et al*. 2008) also suggests, as is so often the case in evolution, that a substantial part of the architecture necessary for the evolution of the nervous system has evolved ‘in advance’. Even more significantly not only do a significant number of the genes, notably those connected with the transport of synaptic vesicles, occur in both plant and yeast but in defining five functional categories associated with neural development in the planarian flatworms, all were also identified in these non-animal groups (Mineta *et al*. 2003).

It is also important to stress that other key components of the nervous system evolved long before even the appearance of sponges. Among the most striking is the very widespread employment of acetylcholine (Wessler *et al*. 2001), notably in plants where, for example, the concentrations of this molecule in rapidly growing tips of bamboo can be 80 times that found in rat brain (Kawashima *et al*. 2007). Intriguingly, the corresponding acetylcholinesterase found in maize appears to be unrelated to the equivalent enzyme in animals (Sagane *et al*. 2005). Such a similarity should not surprise us because in this case the function is based on the well-known serine catalytic triad, which itself is rampantly convergent (e.g. Dodson & Wlodawer 1998; Gheradini *et al*. 2007). Nor is acetylcholine restricted to multicellular organisms, because it also occurs in such protistans as the ciliates (e.g. Delmonte Corrado *et al*. 2001), as do a variety of other hormone-like molecules, including *β*-endorphins and serotonin (e.g. Csaba *et al*. 2007).

Not only are significant parts of the molecular substrate necessary for a nervous system already in position, to be recruited as appropriate, but other key elements of the nervous system have also evolved independently. Most notable in this respect are the Na^+^ voltage-gated channels, which in contrast to the K^+^ and Ca^++^ equivalents are usually thought to be restricted to the animals. Certainly, in this kingdom, there has been independent diversification of the Na^+^ channels (Goldin 2002), but more significantly, such a channel has not only evolved independently in the protistans (Febvre-Chevalier *et al*. 1986) but even in the bacteria (e.g. Ito *et al*. 2004; Koishi *et al*. 2004). It seems, therefore, that the emergence of a nervous system is by no means as improbable as might be thought, and it is equally telling that the evolutionary history of neural evolution is strewn with convergences ranging from the independent evolution of myelination (e.g. Pereyra & Roots 1988; Davis *et al*. 1999) to cognitive landscapes, notably among the corvids (Emery & Clayton 2004; Lefebvre *et al*. 2004). And it is at this point that evolutionary convergences are the most important, but where I cease this review.

## 4. Conclusion

Elsewhere, I have argued that something very like a human is an evolutionary inevitability (Conway Morris 2003), a view that hardly sits comfortably with neo-Darwinian orthodoxy. Here, I have tried to show in the most sketchy manner how major transitions are unproblematic and that paradoxically unrelenting divergence is always accompanied by convergence. It also seems that there are a number of additional strands that collectively paint a heterodox picture of evolution and so are worthy of further exploration. It is, for example, striking how frequently complex systems emerge on the basis of a pre-existing molecular architecture. In addition, although under-appreciated, the emergence of major groups often displays a remarkably mosaic-style of evolution. Here, ostensibly ‘key’ characters, which subsequently often serve to define monophyletic assemblages, show a seemingly erratic and ‘unexpected’ set of distributions. In many cases, we also see that the particular molecules show a remarkable versatility of function in what appear to be unrelated contexts. It is most probable that these molecules are homologous, but in many cases the overall architecture and the iron constraints of active sites (or equivalents) suggest that convergence should not be automatically dismissed. It is also striking how in general the idea that primitive groups are simple, almost skeletal constructions in comparison to their descendants, is simply incorrect and in precursors as diverse as the last common ancestor of the eukaryotes or choanoflagellates we either infer or see an extraordinary degree of complexity. Rather than imagining that this arose by a series of conveniently cryptic prior stages, we may have to face the possibility that evolution involves what to us seem to be a baffling series of self-organizations.

Evolution is no stranger to heterodoxy, but it is striking how the Darwinian mantra continues to strangle innovation. Could we begin to accept that Darwin got it right in terms of mechanism, but frankly this is as interesting as ionic bonding would be to most chemists (other than those who choose to study it). What we do not understand is how organisms assemble as exceedingly complex functional entities nor why they repeatedly navigate to convergent solutions. What we can be sure of, I suggest, is that these processes are predictable, and that this should provide modest encouragement that there is still work to be done.
